# Night Shift Work Is Associated with Reduced Rate of Humoral Response Following Vaccination for HBV

**DOI:** 10.3390/ijerph19148834

**Published:** 2022-07-20

**Authors:** Luca Coppeta, Cristiana Ferrari, Marco Trabucco Aurilio, Gianluigi Ferrazza, Andrea Magrini, Stefano Rizza

**Affiliations:** 1 Department of Biomedicine and Prevention, University of Rome Tor Vergata, 00133 Rome, Italy; luca.coppeta@ptvonline.it (L.C.); andrea.magrini@uniroma2.it (A.M.); 2 School of Occupational Medicine, University of Rome Tor Vergata, 00133 Rome, Italy; 3 Department of Medicine and Health Sciences “V. Tiberio”, University of Molise, 86100 Campobasso, Italy; marco.trabuccoaurilio@unimol.it; 4 Department of System Medicine, University of Rome Tor Vergata, 00133 Rome, Italy; gianluigi.ferrazza@ptvonline.it (G.F.); stefano.rizza@uniroma2.it (S.R.)

**Keywords:** HBV, healthcare workers, vaccination, night shift, shift work, immunological memory

## Abstract

Night shift work has been associated with cardiovascular and metabolic disease, endocrine and immunological disorders. Published studies have reported that a reduced total sleep time with sleep-wake cycle alterations were associated with a reduced rate of humoral response following vaccination. Our study aimed to evaluate the association between night shift work and serological status for HBV among workers employed in a university hospital in Rome. We evaluated medical records of 986 HCWs working at Tor Vergata Policlinic of Rome. We screened all study subjects for anti-HBs IgG, anti-HBc IgG and HBsAg. Serological protection for HBV was evaluated in relation to sex, age group, job task, risk setting and night shift work status. Protective titer was found in 856 (86.8%) study participants and the mean titer was significantly high in females, in subjects aged less than 40 years, in night shift workers and in high-risk setting workers. After adjustment for study covariates, night shift work was no longer associated with an HBV-protective titer. This finding suggests that a vaccination strategy for dampening HBV transmission should be carefully addressed in health care workers (HCWs) doing night shift.

## 1. Introduction

Immunity and sleep are bidirectionally connected. Immune system activation alters sleep, which in turn influences innate and adaptive immunity. Therefore, regular sleep promotes a correct immune response following antigenic stimulus since the immune system processes are organized according to circadian rhythms. Moreover, infections affect sleep regulation, most likely via proinflammatory cytokine production [1], and similarly, chronic sleep deprivation or sleep disruption increase the risk of severe chronic systemic diseases that have a low-grade inflammatory component, like diabetes, atherosclerosis, and neurodegeneration [2].

Night shift work (NSW) is highly prevalent in Western societies, with up to 20% of the European working population engaged in some type of shift work schedule, increasing to 45% in the healthcare sector [3]. However, overall shift work can disturb the worker’s circadian rhythm, thus affecting sleep quality, in particular if employees work night shifts. In fact, night shift workers’ behavioral and environmental cycles are typically misaligned relative to the endogenous circadian system. Therefore, compared with day workers, NSWs have an increased risk of cardiovascular and metabolic disease [4,5,6,7], but, interestingly, recent published studies have also reported that NSWs have slight immunological disorders, such as unbalanced levels of circulating monocytes, T-lymphocytes and immunological biomarkers [8,9]. However, the impact of circadian rhythm disruption on the persistence of immune response following vaccination remains unclear [10]. Recently, we confirmed [11] previous evidence [12,13] that the duration of immunity evidenced by the anti-HB’s titer at the time of the first employment is related to the age of vaccination, confirming that the maturity of the immune system plays a central role in the primary response to vaccination and in the persistence of immune memory.

This point is particularly important because HCWs are at a higher risk of HBV infection than the general population, likely due to the particular characteristics of the health care sector [14,15,16,17,18,19]. Moreover, infected HCWs can also transmit their infection to susceptible patients [20] and the risk for HBV seroconversion following percutaneous exposure to infected blood is particularly high [21,22,23]. Accordingly, HBV vaccination for susceptible HCWs is strongly recommended [24,25,26]. Therefore, because the effectiveness and the duration of immunity after HBV vaccination is a crucial element for the safety of HCWs, we conducted a hypothesis-generating, cross-sectional analysis of clinical, working and HBV serological characteristics of a group of HBV-vaccinated HCWs recruited from the same working environment.

## 2. Materials and Methods

The study was a cross-sectional, hospital-based survey conducted by the Tor Vergata Occupational Medicine Service in 2018 and approved by the Independent Ethics Committee of the University Hospital PTV (Policlinico Tor Vergata) in Rome, Italy. Participants were eligible if they had been working for a minimum of 2 years. The exclusion criteria included a diagnosis of diabetes, liver disease, renal insufficiency, heart failure, coagulopathy, a history of any form of cancer and positive blood tests for HIV. We also excluded HCWs who tested positive for HBs Ag because they were considered being affected by chronic HBV hepatitis and excluded operators who were HBs-Ag negative but positive to anti-HBc-IgG because they have had a previous infection.

Participants who tested negative both for HBs-Ag and anti-HBc-IgG were included. Moreover, we excluded unvaccinated HCWs from the analysis (*n* = 4). Since the vaccination certificate was not available for all study subjects, we divided participants in two groups according to their anti-HBs antibodies level, as follows:

1. subjects with HBs IgG levels ≥ 10 mIU/mL were considered serologically protected;
2. subjects with HBs IgG levels < 10 mIU/mL were considered serologically unprotected (participants with a weak response to vaccination).

We also provided information regarding the time elapsed between the administration of the last dose of HBV vaccination, which was reported by each study participant, and the date of serological evaluation of HBs IgG levels.

The participants received detailed information about the study protocol, and, after providing written consent (Aut. N. 24537/18), they underwent clinical examination. For the study protocol, we analyzed the following covariates: age, sex, smoke, time from the last HBV vaccination, anti-HBs IgG, anti-HBc IgG level and HBsAg status. Moreover, we grouped participants according to the type of work (nurses vs. other types of workers such as physicians and technicians). We also divided participants into two groups: (1) Night shift workers (NSWs), working a shift schedule of four to seven 12 h nights per month, followed by 2 days off, and (2) day workers who had never worked night shifts. Finally, we defined HCWs as being at high-risk of HBV infection if they worked in departments at the frontline of diagnosis, treatment, and care of high-risk patients (high-risk departments (infectious disease, pneumology, internal medicine, and emergency room) vs low-risk departments (others)).

Serological evaluation was performed by means of the ECLIA (Electro Chemi Luminescence Immuno Assay) method.

### Statistical Analyses

Participants’ clinical characteristics are reported as numbers and percentages for categorical variables. The significance of the difference in percentages between groups is evaluated with the χ^2^ test or Fisher’s Exact Test, when appropriated. Finally, we built up a simple and multiple logistic regression analysis with forward method to explore independent associations between HBs-IgG-titer (protective vs not protective) and possible predictors significantly correlated with dependent variable. A *p* value < 0.05 was considered statistically significant. All analyses were performed in IBM SPSS version 25.0 for Windows.

## 3. Results

We screened 1013 health care hospital employees. After screening, we excluded 11 individuals for positive HBs Ag; six out of eleven were born in foreign countries (two in Africa, three in Albania and one in Romania). None of those subjects had a documented vaccination history. We also excluded 13 HCWs because they tested positive for HBs Ag and anti-HBc. Of these, eight HCWs were born in Italy and five were born after 1980 (in 1991, a law was passed mandating the universal vaccination of infants and 12-year-old adolescents. Moreover, we excluded two individuals for renal insufficiency and one for overt diabetes. The study finally included 986 participants (556 (56.4%) nurses and 430 (43.6%) other tasks).

The mean age of the study participants was 40.7 ± 9.29 years old. As shown in Table 1, the HCWs aged older than 40 years old were 52% of the study population. The majority of participants were females and night shift workers. However, they did not differ with respect to age class, smoke, job task and hospital risk setting status. Table 2 shows the study characteristics of the HCW population according to HBV titers after vaccination. The group of HCWs aged older than 40-years old had a significant higher HBV-protected titer than those aged under 40 (Table 2, *p* > 0.01). HBV-protected HCWs were more frequently females and working in a hospital’s high-risk setting. Compared with HBV-unprotected HCWs, protected HCWs had a significantly reduced time between the administration the last dose of HBV vaccination and the serological evaluation of HBs IgG level. Moreover, we found a significant negative correlation between HBV IgG level and the months elapsed from the administration of the last dose of HBV vaccination (r = −0.097, *p* = 0.02).

Finally, using a logistic regression model, we found that NSWs had a 1.42 times not significant odds (95% CI 0.853–2.144, *p* = 0.193) of a protective HBV titer compared with day workers, when adjusted for covariates (Table 3).

## 4. Discussion

Healthcare workers are at risk of occupational needle-stick injuries, such as HBV infection. Therefore, HBV vaccination for susceptible HCWs is highly recommended. However, although through univariate analysis we found that NSWs had a significant tendency toward a higher rate of protective anti-HBs titer than day-workers, this difference was no longer significant after controlling for sex, age class, months from last HBV vaccination, hospital job task and high-risk settings.

Despite its economic and social relevance, shift work results in a disruption of biological rhythms, thus predisposing individuals to poor metabolic and cardiovascular health in response to reduced sleep. In fact, the literature indicates that NSWs are often affected from cardio-metabolic disorders [6,7,27,28] due to unhealthy behavioral factors, such as hyper-caloric diet, sedentarism and obesity. Accordingly, we have recently reported that HCWs with a “dysfunctional circadian clock” and with increased levels of IL1b carry a higher risk of developing carotid atherosclerosis through an overload of IL6 being released [29]. Moreover, it is well known that the activity of the immune system is coordinated by intrinsic molecular clocks in blood T and B lymphocytes, monocytes, macrophages and other inflammatory cells [30,31,32] and the expression of genes regulating immune responses can be modulated by alteration of the circadian rhythm [33,34,35]. Accordingly, protracted shift work has been linked to increased risk and severity of autoimmune disorders [30]. Therefore, although the main finding of this work is a simple association, even if controlled for significant confounding factors, it is intriguing to speculate that the night shift work may influence the immune response to HBV with respect to a deficient function of immune cells. It is possible that immune cells of NSWs, influenced by an overload of inflammatory mediators due to a “dysfunctional circadian clock”, lose earlier their immunocompetence as well as the persistence of immune response following vaccination. However, this speculation is in keeping with our recent finding [5]. In this paper, we demonstrated that the REV-ERBα and BMAL1 gene expression in peripheral blood mononuclear cells (PBMCs), a surrogate of the molecular clock, being unbalanced earlier in NSWs is probably caused by aberrant and prolonged artificial light exposure during night shifts. Unfortunately, we have no data to show regarding functional indexes of immune system of these study participants. However, although our study results indicated that HCWs with HBs IgG levels ≥ 10 mIU/mL were vaccinated for a longer time than HCWs with HBs IgG levels < 10 mIU/mL, recent studies clearly showed that a significant percentage of HCWs, having an anti-HBs titer below the cut-off value of 10 mIU/mL, should be considered protected. In fact, although we could not provide valid information regarding the number of vaccine doses that study participants received, serological response after the administration of a booster dose of vaccine suggests that an immunological memory persists even if anti-HBs titer decreases under the protective level [15,16].

Regarding other factors evaluated in the present study, we found that low protection levels for HBV are related to age classes of 40-years or older, according to the lower vaccine coverage rate (in 1991, Italy implemented a mandatory HBV vaccination program; through the program, which aimed to immunize all newborns and children aged 12 years, more than 12 million children were vaccinated against HBV) and the physiological decrease of anti-HBs titer over time following primary vaccination cycle [36]. We also reported a significantly higher protection level among nurses in comparison with other job tasks; this result, in our opinion, is due to the compulsory vaccination for nursing school entry, while it is only highly recommended for other healthcare professionals [24,25,26].

Finally, in our study, females HCWs have had significantly higher HBV IgG titer than male participants. Although these data need to be confirmed in larger cohorts, it is known that sex significantly affects the competence of immune responses, contributing to differences in the pathogenesis of infectious diseases. Females usually evolve with more intense immune responses to viral infections and to vaccination in comparison to male individuals, because, typically, estrogens promote an immune-stimulating effect, while androgens are immune-suppressing [37,38,39,40].

## 5. Conclusions

Our investigation offers additional data on the association between occupational factors and HBV status among HCWs. However, while there are many open questions regarding longitudinal efficacy and the mechanisms underlying the effects of vaccination on HCWs working night shifts, future studies are warranted to understand which type of appropriate strategies aimed at improving the effectiveness and the duration of immunity after HBV vaccination may help in night shift workers.

## Figures and Tables

**Table 1:** General characteristics of study population.

	*n*	Percent
*Total number*	986	
*Mean age (SD)*	40.7 ± 9.3	
*Mean Titer*	400.5 ± 297.7	
*Months from last HBV vaccination*	148.0 ± 64.6	
**Sex**		
*Male*	288	29.2
*Female*	698	70.8
**Smoke**		
*Active smokers*	360	36.5
*Former or never smokers*	626	63.5
**Age class**		
*<40 years*	474	48.1
*≥40 years*	512	51.9
**Job task**		
*Nurse*	556	56.4
*Other tasks*	430	43.6
**Night shift**		
*Yes*	752	76.3
*No*	234	2..7
**High-risk setting**		
*Yes*	482	51.1
*No*	504	48.9

**Table 2:** Main characteristics of the study population according to HBV titer.

Variables	Subjects with HBs IgG Levels ≥ 10 mIU/mL (*n* = 856)	Subjects with HBs IgG Levels < 10 mIU/mL (*n* = 126)	*p*
Sex (male/female)	228/628 (26.6/73.4)	58/68 (46.0/54.0)	<0.01
Smokers (active/former or never)	349/507	58/68	0.295
Months between the last HBV vaccination and the dosage of HBs IgG levels (days).	144.5 ± 60.4	171.3 ± 83.7	<0.01
Age class (<40 years/≥40 years)	436/420 (50.9/49.1)	37/89 (29.3/70.7)	<0.01
Job task (nurse/other tasks)	492/364 (57.5/42.5)	60/66 < (47.6/52.4)	<0.05
Night shift workers (yes/no)	664/192 (77.5/22.5)	85/41 (67.4/32.6)	<0.01
High-risk setting (yes/no)	438/418 (51.2/48.8)	43/83 (34.1/65.9)	<0.01

**Table 3:** Multivariate logistic regression model with HBs IgG levels ≥ 10 mIU/mL as a dependent variable.

Variables	ODDs Ratio (OR)	95%CI for OR	*p*
Sex (male)	0.479	0.310–0.699	<0.001
Age class (≥40 years)	0.299	0.185–0.478	<0.001
Job task (nurse)	2.388	1.498–3.679	<0.001
Months from HBV vaccination	0.994	0.991–0.997	<0.001
Night shift workers	1.421	0.853–2.144	0.193
High-risk setting	2.145	0.656–6.699	0.213
